# Interleukin (IL)-6: A Friend or Foe of Pregnancy and Parturition? Evidence From Functional Studies in Fetal Membrane Cells

**DOI:** 10.3389/fphys.2020.00891

**Published:** 2020-07-24

**Authors:** Chasey Omere, Lauren Richardson, George R. Saade, Elizabeth A. Bonney, Talar Kechichian, Ramkumar Menon

**Affiliations:** ^1^Division of Maternal-Fetal Medicine and Perinatal Research, Department of Obstetrics & Gynecology, The University of Texas Medical Branch at Galveston, Galveston, TX, United States; ^2^Department of Obstetrics, Gynecology and Reproductive Sciences, College of Medicine, The University of Vermont, Burlington, VT, United States

**Keywords:** amniotic epithelial cells (AECs), cytokines, EMT, inflammation, fetal membranes

## Abstract

**Objective:**

Protection of the fetus within the amniotic sac is primarily attained by remodeling fetal membrane (amniochorion) cells through cyclic epithelial to mesenchymal and mesenchymal to epithelial (EMT and MET) transitions. Endocrine and paracrine factors regulate EMT and MET during pregnancy. At term, increased oxidative stress forces a terminal state of EMT and inflammation, predisposing to membrane weakening and rupture. IL-6 is a constitutively expressed cytokine during gestation, but it is elevated in term and preterm births. Therefore, we tested the hypothesis that IL-6 can determine the fate of amnion membrane cells and that pathologic levels of IL-6 can cause a terminal state of EMT and inflammation, leading to adverse pregnancy outcomes.

**Methods:**

Primary amnion epithelial cells (AECs) were treated with recombinant IL-6 (330, 1,650, 3,330, and 16,000 pg/ml) for 48 h (*N* = 5). IL-6-induced cell senescence (aging), cell death (apoptosis and necrosis), and cell cycle changes were studied using flow cytometry. Cellular transitions were determined by immunocytochemistry and western blot analysis, while IL-6 signaling (activation of signaling kinases) was measured by immunoassay. Inflammatory marker matrix metalloproteinase (MMP9) and granulocyte-macrophage colony-stimulating factor (GM-CSF) concentrations were measured using a Fluorokine E assay and ELISA, respectively. Amniotic membranes collected on gestational day (D) 12 and D18 from IL-6 knockout (KO) and control C57BL/6 mice (*N* = 3 each) were used to determine the impact of IL-6 on cell transitions. Fold changes were measured based on the mean of each group.

**Results:**

IL-6 treatment of AECs at physiologic or pathologic doses increased JNK and p38MAPK activation; however, the activation of signals did not cause changes in AEC cell cycle, cellular senescence, apoptosis, necrosis, cellular transitions, or inflammation (MMP9 and GM-CSF) compared to control. EMT markers were higher on D18 compared to D12 regardless of IL-6 status in the mouse amniotic sac.

**Conclusion:**

Physiologic and pathologic concentrations of IL-6 did not cause amnion cell aging, cell death, cellular transitions, or inflammation. IL-6 may function to maintain cellular homeostasis throughout gestation in fetal membrane cells. Although IL-6 is a good biomarker for adverse pregnancies, it is not an indicator of an underlying pathological mechanism in membrane cells.

## Introduction

Human fetal membranes (amniochorion) are the innermost lining providing structure as well as immune and mechanical protection to the uterine cavity (Menon, 2016). Membranes start their growth at embryogenesis as two independent layers, i.e., a single layer of amnion epithelium and multilayered chorion trophoblast, which fuse to form the amniochorion, demarcated by a collagen-rich extracellular matrix (ECM) by the early second trimester of pregnancy (Strauss, 2013). Mesenchymal cells are disbursed throughout the ECM and form a key cellular component of the membrane (Strauss, 2013).

During pregnancy, the maintenance of membrane structural integrity is critical to withstand the stretch and stress imposed by the growing volume of the uterine cavity. Highly elastic amnion layer membranes maintain homeostasis through cyclic cellular transitions of amnion epithelial cells (AEC) to amnion mesenchymal cell (AMC) (EMT) and mesenchymal back to epithelial (MET) cells (Richardson et al., 2020). A terminal state of EMT occurs at term where MET is stalled due to oxidative stress (OS)-induced senescence (Richardson et al., 2020) and accumulation of pro-EMT factors like TGFβ in membrane cells and in the amniotic fluid (Richardson et al., 2018). This leads to the accumulation of AMCs in the matrix and promotes localized inflammation and collagenolysis, leading to mechanical weakening (Richardson et al., 2020). This is partly because compared to AECs, AMCs are much more vulnerable to ROS and produce inflammatory mediators (Ko et al., 2012; Denu and Hematti, 2016). Overwhelming membrane inflammation and the propagation of inflammatory mediators to quiescent uterine tissues can transition a quiescent cervix and myometrium into a pro-labor active state (Sheller-Miller et al., 2016; Hadley et al., 2018). Fetal membranes, therefore, play a major role in pregnancy maintenance as well as in promoting parturition. Understanding the factors that maintain membrane cellular, structural, functional, and mechanical integrity is therefore important.

Membranes are rich sources of several biochemical mediators that help to maintain various membrane functions (Menon et al., 2015), although their precise contributions are unclear (Feng et al., 2018). Knowledge of these functional contributions is critical to understanding the physiologic and pathologic contribution of these biochemicals during pregnancy and in term and preterm parturitions. This study is an attempt to understand the functional role of interleukin (IL)-6 in amnion membrane functions. IL-6 is a pleiotropic cytokine that has been documented to perform distinct functions during various stages of pregnancy, including implantation, embryogenesis, pregnancy, and parturition (Prins et al., 2012). Infection and injuries to tissues can increase IL-6 production and generate a host inflammatory response by stimulating acute phase responses (Prins et al., 2012). IL-6 can also control inflammation by minimizing the impact of other inflammatory cytokines such as IL-1β and TNF-α (Prins et al., 2012). IL-6 is produced by human fetal membranes, and its expression increases in both amnion and chorion cells in response to infectious stimuli (Santhanam et al., 1991; Fortunato et al., 1994; Snyers and Content, 1994; Menon et al., 1995; Keelan et al., 1997). IL-6 is also one of the most studied biomarkers in spontaneous preterm birth (PTB) and preterm prelabor rupture of the membrane (pPROM) (Santhanam et al., 1991; Menon et al., 2011). It has been reported that the concentration of IL-6 is increased in the amniotic fluid (Romero et al., 1990), cervical vaginal fluid (Lockwood et al., 1994, 2010), fetal membranes (Santhanam et al., 1991), decidua (Lockwood et al., 2010), myometrium (Rauk et al., 2001), and cervix in PTB and pPROM (Chai et al., 2012), both in cases of microbial invasion of the intraamniotic cavity (MIAC) as well as in the absence of infection (Prins et al., 2012). Recently, many investigators have proposed IL-6 as a predictor of MIAC and intraamniotic inflammation (IAI) in PTB and pPROM (Chaemsaithong et al., 2015, 2016a,b; Kacerovsky et al., 2018). IL-6 is also a member of the senescence-associated secretory phenotype (SASP) family, and IL-6 expression is higher in fetal membranes at term labor compared to term not in labor (Menon et al., 2016). Functionally, the IL-6 knockout model has been shown to lead to delayed term delivery (Robertson et al., 2010), suggesting a pro-parturient function; however, several *in vitro* human cell/tissue-based studies (Mitchell et al., 1991; Kent et al., 1993; Lockwood et al., 2010; Devi et al., 2015) and non-human primate studies (Sadowsky et al., 2006) create ambiguity regarding its exact functional role in promoting parturition either at term or preterm.

IL-6 has also been reported to promote cellular proliferation (Lee et al., 2016) and migration (Jovanovic and Vicovac, 2009), EMT (Lee et al., 2016; Xiao et al., 2017; Browning et al., 2018; Sun et al., 2018), as well as senescence (Kojima et al., 2013). We have earlier reported that human fetal membrane cells, specifically AECs, undergo proliferation, migration, and transitions during pregnancy and aging at term (Richardson and Menon, 2018). However, reported roles of IL-6 are rather vague and this ambiguity regarding its functional role during pregnancy and parturition led us to conduct multiple functional tests in fetal membrane cells. It is likely that IL-6 may play multiple functional roles in regulating membrane homeostasis during gestation or in the promotion of senescence at term. Using an *in vitro* model of primary AECs, we tested proliferation and the cell cycle, cellular aging (senescence), cell death (necrosis and apoptosis), cellular transitions, cell signaling, and the generation of inflammation in response to physiologic (term pregnancy, not in labor and labor) and pathologic [spontaneous preterm birth (PTB) and pPROM with MIAC and intraamniotic inflammation (IAI)] concentrations of IL-6 seen in the amniotic fluid.

## Materials and Methods

### IRB Approval

This study protocol was approved by the Institutional Review Board at The University of Texas Medical Branch (UTMB) at Galveston, TX, United States, as an exempt protocol to use discarded placenta after normal term cesarean deliveries (UTMB 11-251). No subject recruitment or consenting was done for this study and no identifiers were collected.

### Clinical Samples

Samples we collected from the discarded placentas of term deliveries. Fetal membranes were dissected from the placenta, washed three times in normal saline, and cleansed of blood clots using cotton gauze. Tissue was then processes as described below to isolate fetal membrane cells.

#### Term Sample Criteria

Placentas from women (18–40 years old) undergoing elective repeat cesarean delivery (between 37 and 41 weeks of gestation) prior to the onset of labor were included in the study. Women with a prior history of preterm labor and delivery, preterm premature rupture of the membranes, preeclampsia, placental abruption, intrauterine growth restriction, and gestational diabetes were excluded. Patients that were group B *Streptococcus* carriers, who were treated for urinary tract infection, sexually transmitted diseases, chronic infections like HIV, hepatitis, and women who smoked cigarettes or reported drug and alcohol abuse were also excluded from this experiment.

### Isolation and Culture of Human Amnion Epithelial Cells (AECs)

All reagents and media were warmed to 37°C prior to use. The amnion membrane was manually peeled from normal, term, not in labor cesarean section placentas, then rinsed in saline and transferred to a Petri dish that contained Hanks Balanced Salt Solution (HBSS) (Mediatech Inc., Manassas, VA, United States). The amnion membrane was then cut into 2 cm × 2 cm pieces. They were digested twice in 0.25% trypsin and 0.125% collagenase A (Sigma-Aldrich, St. Louis, MO, United States) in HBSS for 35 min at 37°C. After each digestion, the tissue was filtered through a 70 μm strainer (Fisher Scientific, Waltham, MA, United States) cell and trypsin was inactivated using complete Dulbecco’s Modified Eagle’s Medium: Nutrient Mixture F-12 media (DMEM/F12) (Mediatech Inc.) supplemented with 15% fetal bovine serum (FBS) (Sigma-Aldrich), 10% penicillin/streptomycin, 10% amphotericin B (Mediatech Inc.), and 50 μg/mL epidermal growth factor (EGF) (Sigma-Aldrich). The collected filtrate was centrifuged for 10 min at 3,000 *g*. The cell pellet was re-suspended in 5 mL complete DMEM/F12. The cells were then counted using a hemocytometer. Once cells were counted, approximately 3–5 million cells per flask were cultured in T75 flasks containing complete DMEM/F12 media at 37°C, 5% CO_2_, and 95% air humidity until they were 80–90% confluent.

### AEC Treatment With IL-6

Once the passage zero cells were 80–90% confluent, they were passed into a variety of culture plates depending on the assay and allowed to attach as passage one cells for 12–24 h. After cell were attached by 24 h, cells were treated one time 48-h treatments of IL-6 (330, 1,650, 3,300, and 16,000 pg/mL) or control media (Santhanam et al., 1991). The doses of IL-6 were based on reported concentration ranges during various stages of gestation (330–1,650 pg/mL), term labor (3,300 pg/mL), and preterm labor with and without rupture (16,000 pg/mL) with documented MIAC and inflammation (Musilova et al., 2015). After 48 h cells were collected for a variety of end point assays described below.

### Decidua Cell Culture and Validation of Functionality of Recombinant IL-6

Separation of primary decidua cells from the chorio-decidua membrane involved blunt dissection with forceps and a scalpel. The decidua was minced by cross-cutting with scalpel blades. Tissues were processed in a digestion buffer containing 0.125% trypsin (Cat# 85450c, Sigma), 0.2% collagenase (Cat# C0130, Sigma), and 0.02% DNase I (Cat# DN25, Sigma) and incubated at 37°C for 60–90 min. Samples were subsequently neutralized with complete medium (1:1 mixture of Ham’s F12/DMEM, supplemented with 5% heat-inactivated FBS, 10 ng/mL EGF, 100 U/ml penicillin G, and 100 mg/mL streptomycin) (Cat# 30-001-CI, Corning). After filtration, the cell solution was centrifuged at 3,000 rpm for 10 min. A cell-separation gradient was prepared using an Optiprep column (Axis-Shield), with steps ranging from 4 to 40% of 4 mL each (4, 6, 8, 10, 20, 30, and 40%). Processed decidual cells were added to the top of the gradient and centrifuged (3,000 × *g*) at room temperature for 35 min. Cell densities of 1.027–1.038 g/mL represented the decidua layer. Harvested cells were washed with DMEM, centrifuged, resuspended in DMEM, and plated at a density of 80,000 (decidua) per well to yield cultures with 95–99% purity. Decidua cells were allowed to attach for 12–24 h and then treated with various concentrations of IL-6 for 12. After 48 h of incubation, the cells were lysed and stained for STAT3 by western blot to confirm IL-6 potency (Devi et al., 2015) (Supplementary Figure S1).

### Multiplex Assay for Protein Kinases

Medium that was collected from the IL-6 treated cells were used in the Milliplex MAP cell signaling buffer and detection kit for magnetic beads (Millipore, Burlington, MA, United States) (i.e., Phospho JNK, ERK, p38, ATF2, MSK1, c-JUN, STAT1, and HSP27) (*N* = 5). Media and substrates were added to the kit plate and instructions were followed per kit protocol. Briefly, assay buffer was added to the plate, then removed. Then, magnetic beads, assay buffer, and sample lysates were added to the plate and were incubated for 16–20 h at 4°C on a shaker in the dark. The buffer was then removed and the beads were washed twice using assay buffer. 1X Milliplex MAP detection antibody was added to the plate and incubated on a shaker for 1 h at room temperature (20–25°C) in the dark. The detection antibody was then removed, and 1X streptavidin-PE was added and incubated for 15 min on the plate shaker in the dark. Amplification buffer was then added. The plate was run using a Luminex 200 (LX200-XPON-IVD, Luminex Corporation, Austin, TX, United States) apparatus. Data was then analyzed.

### Flow Cytometry Assays

#### Cell Cycle Analysis

Cells were plated in 12-well plates, with 30,000 cells per well (*N* = 3). The cells were left to attach for 12–24 h after the cells were plated, then they were treated with IL-6 at different concentrations for 48 h. Cells were collected by adding trypsin, then trypsinization was stopped using complete medium. Cells were then transferred to a conical tube. Cells were centrifuged at 2,000 *g* for 5 min. A cell cycle assay kit (c03551, Beckman Coulter, Brea, CA, United States) was used. Propidium iodine were added to the cells, and they were run on a flow cytometer.

#### AEC Senescence Flow

Cells were plated in 6-well plates, with 300,000 cells per well (*N* = 3). The cells were left to attach for 12–24 h after the cells were plated, then they were treated with IL-6 at different concentrations. At the 48-h endpoint, the medium was removed. Cells were treated with diluted bafilomycin (cat# BML-CM110-0100, Enzo Life Sciences, Farmingdale, NY, United States) and the negative control was treated with DMSO. Cells were incubated at 37°C for 1 h. C12FDG (cat#7188 Setareh Biotech, Eugene, OR, United States) was added to the cells, except for the negative control. Cells were collected by adding trypsin, then trypsinization was stopped using complete medium. Cell were then transferred to a conical tube and centrifuged at 3,000 *g* for 10 min. The medium was removed and the pellet was re-suspended in 300 μL of annexin buffer treated with propidium iodine. The cells were run on a flow cytometer using a standard senescence-associated-β-galactosidase template (SA-β-Gal).

#### AEC Apoptosis and Necrosis

Cells were plated in 12-well plates, with 30,000 cells per well (*N* = 3). The cells were left to attach for 12–24 h after the cells were plated, then they were treated with IL-6 at different concentrations for 48 h. At the 48-h endpoint, the medium was removed. Cells were collected by adding trypsin, then trypsinization was stopped using complete medium. Cell were then transferred to a conical tube and centrifuged at 2,000 × *g* for 5 min. The medium was removed and the pellets were re-suspended in 100 μL of annexin buffer and treated with propidium iodide and Alexa Fluor 488 annexin-V from the Alexa Fluor 488 Annexin-V/Dead 1 Kit (V13241, Fisher Scientific, Hampton, NH, United States). The cells were run on a flow cytometer; Q2-UR represents necrotic cells and Q2-LR represents apoptotic cells.

### Microscopy

#### Brightfield Microscopy

Brightfield microscopy images were captured using a Nikon Eclipse TS100 microscope (4, 10, and 20×) (Nikon, Melville, NY, United States). Three regions of interest per condition were used to determine the overall cell morphology.

#### Fluorescence Microscopy

Fluorescent microscopy images were captured using a Keyence All-in-one Fluorescence BZ-X810 microscope (Keyence Corporation of America, Itasca, IL, United States). Three regions of interest per condition were used to determine the ratio of cytokeratin-18 and vimentin.

### AEC Immunohistochemistry for EMT Markers

Cells were plated in 8-well glass coverslip, with 50,000 cells per well (*N* = 5). The cells were left to attach for 12–24 h after the cells were plated, then they were treated with IL-6 at different concentrations. At the 48-h endpoint, the cells were fixed in 4% PFA, then washed with PBS and 0.5% Triton-X. The cells were blocked using 3% BSA, and primary antibodies were added: cytokeratin-18 (CK-18, 1:800, ab668, Abcam, Inc. Cambridge, MA, United States) and vimentin (1:300, ab92547, Abcam Inc. Cambridge, MA, United States). The cells were then washed with PBS and DAPI was added. The images were then captured using fluorescent microscopy at 20×. Uniform laser intensity and brightness/contrast analysis was conducted on vimentin and CK-18 levels to determine the vimentin/CK-18 ratio (Richardson and Menon, 2018; Richardson et al., 2019, 2020).

### C57/B6 Samples

The IL-6 deficient mice used in this study were previously reported (Poli et al., 1994). Wild type (Jackson Laboratory, Bar Harbor, ME, United States) and IL-6 deficient C57BL/6 mice were housed under AAALAC-approved (IACUC #19-018) and specific pathogen-free conditions using a 12-h light cycle and *ad libitum* food (normal mouse chow) and water. Mice underwent timed mating as previously described (Bonney et al., 2016). Briefly, female mice were seasoned to male pheromones for 3 days and then mated for 18 h although the mating window is typically 4–6 h around midnight, after which the males were removed. Copulatory plugs are checked the following morning (∼18 h later) to confirm mating. Mice are then monitored and a weight gain of >1.75 g by E10 are confirmed pregnant. At D12 and D18 of gestation, pregnant females were euthanized and intrauterine tissues were removed and snap-frozen in liquid nitrogen until tissue lysis was conducted (*N* = 3). After opening the uterus, fetal tissues and underlying implantation uterus were removed en bloc. The amniotic sacs were dissected away from the fetal placental unit.

### Western Blot Analysis

Decidua and mice tissue were lysed with RIPA lysis buffer (50 mM Tris pH 8.0, 150 mM NaCl, 1% Triton X-100, and 1.0 mM EDTA pH 8.0, 0.1% SDS) supplemented with protease and phosphatase inhibitor cocktail and phenylmethylsulphonyl fluoride. After centrifugation at 10,000 rpm for 20 min, the supernatant was collected. Protein quantification was performed using the Pierce BCA protein assay kit (Thermo Fisher Scientific). The protein samples (*N* = 3) were separated using precast gels and transferred to the membrane using an iBlot1 gel transfer device (Thermo Fisher Scientific, Waltham, MA, United States). Membranes were blocked in 5% blotting grade blocker made with 1x Tris buffered saline-Tween 20 (TBS-T) buffer for 1 h. The primary antibody was added and the membrane was left to rock overnight at 4°C. The membrane was incubated with a secondary antibody. For membranes that were stripped, restore western blot stripping buffer was used; none of the membranes in this study were stripped more than three times. The following non-human antibodies were used: vimentin (ab92547, Abcam Inc. Cambridge, MA, United States), actin (Sigma-Aldrich, A5441), and STAT3 (SC8019, Santa Cruz, Waltham, MA, United States).

### Statistical Analysis

Statistical analysis for normally distributed data, as defined by Prisms normality test, was performed using an ANOVA with Tukey’s multiple comparisons test and one-tailed unpaired *t*-tests. Statistical values were calculated using GraphPad Prism; *P*-values less than 0.05 were considered significant. Additionally, fold changes were calculated based on data means. Data are represented as mean ± SEM in bar graphs. All data were analyzed using GraphPad Prism 6.

## Results

### Term Labor IL-6 Concentrations Activate JNK and p38MAPK but Do Not Induce Pro-labor Inflammatory Markers in AECs

To test that the recombinant IL-6 used in our experiments was functionally active, primary decidua cells were treated with a range of IL-6 concentrations and the activation of the downstream transcription factor STAT3 was determined as previously shown by Devi et al. (2015). As expected, IL-6 induced STAT3 expression in these cells, showing their functional viability (Supplementary Figures S1A,B).

**FIGURE 1:**
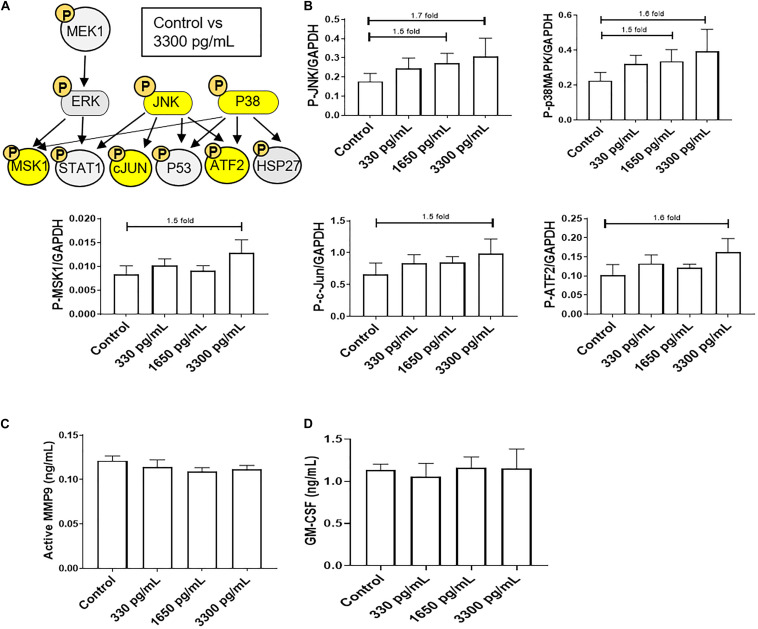
IL-6 dose reported in term labor amniotic fluid activates JNK and p38MAPK, leading to downstream kinase activation in AECs. **(A)** Schematic of various phosphorylated (P) kinases tested using a multiplex immunoassay. Highlighted (yellow) P-proteins were upregulated, though not significantly, after IL-6 treatment mimicking term labor (3,300 pg/mL) compared to control-treated AECs. **(B)** IL-6 treatment (3,300 pg/mL) of AECs showed increasing trends of upstream kinases activation, JNK (1.7-fold) and p38MAPK (1.6-fold), which contributed to the downstream activation of MSK1 (1.5-fold), c-JUN (1.5-fold), and ATF2 (1.5-fold) compared to control samples. *N* = 5; mean ± SEM medium florescent intensity. **(C,D)** IL-6 did not increase the inflammatory profile of AECs, as seen by no changes in MMP9 activation or GM-CSF production when compared to control or term labor IL-6 treatment (3,300 pg/mL). *N* = 5; mean ± SEM.

In order to evaluate the ability of IL-6s to activate various signaling pathways in AECs, a multiplex kinase panel was conducted. Term labor (3,300 pg/mL) IL-6 levels showed increasing trends of upstream kinases JNK and p38MAPK activation compared to control AECs (Figures 1A,B), whereas lower doses had no effect on any of the kinases. Additionally, the downstream kinases MSK1, ATF, and c-Jun showed similar activation trends (Figures 1A,B). However, activation of these kinases did not induce some of their downstream targets such as pro-labor inflammatory mediators MMP9 and GM-CSF (Figures 1C,D) compared to controls, suggesting the high levels of IL-6 do not functionally contribute to the inflammatory onslaught required to induce labor in AECs.

Due to the inability of IL-6 to activate pro-labor kinase pathways leading to inflammation, further experiments were conducted to determine the role of IL-6 in cellular processes in AECs.

### Gestational Concentrations of IL-6 Do Not Induce Changes in the Cell Cycle, Cellular Aging, Cell Death, or Cellular Transition in AECs

IL-6 treatments mimicking early (300 pg/mL), mid (1,650 pg/mL), and term (3,300 pg/mL) gestation were used to determine if IL-6 could induce cell cycle-related changes in AEC, as reported in other systems (Santhanam et al., 1991). Cell cycle analysis by flow cytometry determined that physiologic doses of IL-6 did not induce any changes in G0, G1, S phase, or G2 and compared to control cells (Supplementary Figure S2).

**FIGURE 2:**
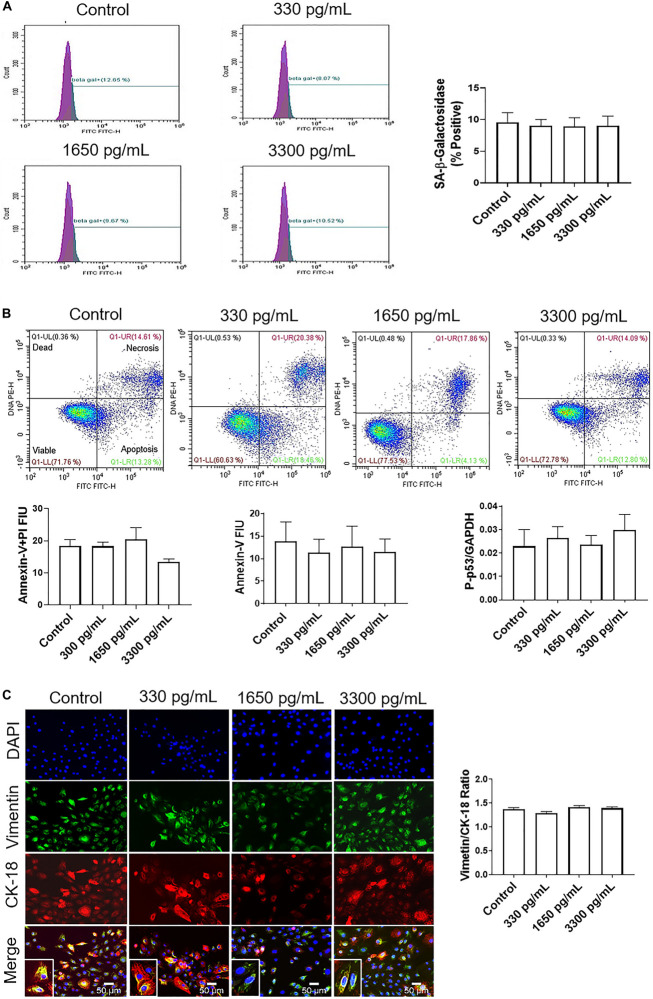
IL-6 at term labor does not induce cellular aging, cell death, or cellular transitions in AECs. **(A)** Flow cytometry analysis of IL-6 induced senescence in AECs. Physiologic concentrations of IL-6 did not increase the population of senescence-associated-β-galactosidase (SA-β-Gal) cells compared to controls. *N* = 5; mean ± SEM. **(B)** Flow cytometry analysis of necrotic and apoptotic cells. Physiologic (330, 1,650, and 3,300 pg/mL) concentrations of IL-6 did not induce necrosis, as indicated by a lack of changes in annexin-V + propidium iodide (PI) positive cells (top right box), nor apoptosis as indicated by no changes in Annexin-V (bottom right box) expression between IL-6 treated and untreated AECs. A protein kinase panel further confirmed the lack of apoptosis by showing that phospho-p53, a pro-apoptotic marker, did not change after treatment with IL-6. Fluorescence intensity units (FIU) *N* = 3; mean ± SEM. **(C)** Immunocytochemistry of intermediate filaments in AEC to assess cell transition. AECs were stained with both cytokeratin-18 (CK-18; epithelial marker) and vimentin (mesenchymal marker) to determine transition status after physiologic (330, 1,650, and 3,300 pg/mL) IL-6 treatments. Uniform intensity analysis was conducted on vimentin and CK-18 levels to derive the vimentin/CK-18 ratio. A high vimentin/CK-18 ratio indicates a mesenchymal phenotype, while a low ratio indicates an epithelial phenotype. IL-6 treatment did not change the vimentin/CK-18 ratio compared to control cells. Fluorescent images were captured at 20×. Red: cytokeratin-18 (CK-18), green: vimentin, blue: DAPI. *N* = 5; mean ± SEM. Scale bar 50 μm.

Regardless of concentration, IL-6 did not induce senescence, as noted by no change in senescence-associated-β-galactosidase (SA-β-Gal) positive cells (Figure 2A). Furthermore, IL-6 did not induce the two main types of cell deaths, i.e., necrosis and apoptosis. As shown in Figure 2B, flow cytometry data showed no change in IL-6-induced necrosis as determined by annexin-V + propidium iodide (PI) negative cells or apoptosis as determined by a lack of annexin-V and P-p53.

Given that our data did not show that IL-6 induced changes to cell cycle, caused cellular aging or cell death, attention was turned to determine if IL-6 induced cellular transitions (EMT or MET) in AEC. AECs were stained with both cytokeratin-18 (CK-18; red; epithelial marker) and vimentin (green; mesenchymal marker) to determine the transition status based on the vimentin/CK-18 ratio after IL-6 treatment (Figure 2C). A high vimentin/CK-18 ratio indicates a mesenchymal phenotype, while a low ratio indicates an epithelial phenotype (Richardson and Menon, 2018; Richardson et al., 2019, 2020). Irrespective of concentration, IL-6 caused a slight change in morphology (see inset images) and the vimentin/CK-18 ratio that kept AECs in a “metastate” (Richardson and Menon, 2018) or native state where they co-expressed both epithelial and mesenchymal markers (Figure 2C).

Based on these data, pathologic doses of IL-6 (16,000 pg/mL) reported in microbial invasion and intraamniotic inflammation were investigated to see if this dose of IL-6 activated pro-labor pathways leading to inflammation.

### IL-6 Concentration Mimicking Infectious and Inflammatory pPROM Activates JNK and p38MAPK but Does Not Induce Pro-labor Inflammatory Markers in AECs

**FIGURE 3:**
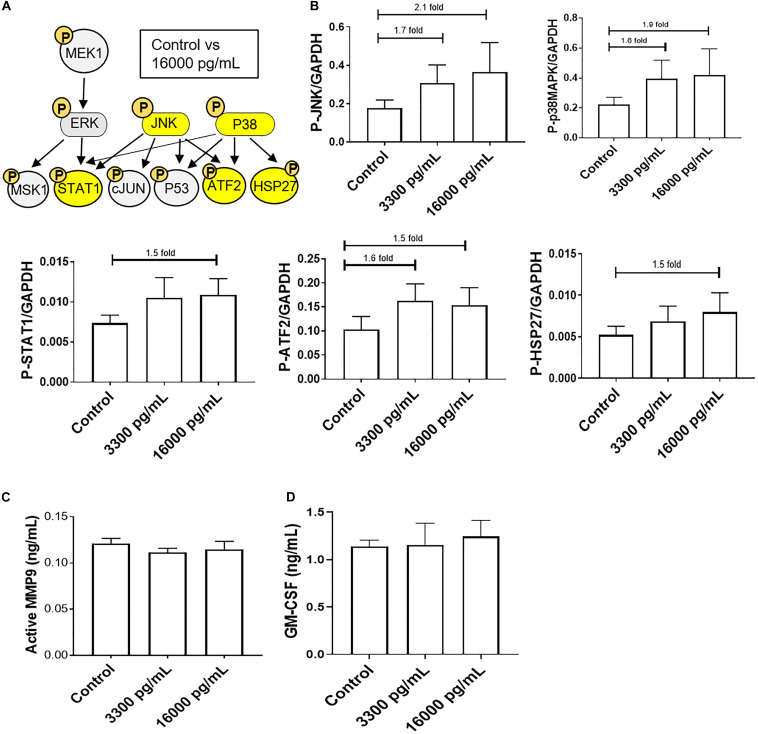
IL-6 concentration reported in the amniotic fluid of infection associated pPROM activates JNK and p38MAPK leading to differential downstream kinase activation in AECs. **(A)** Schematic of various phosphorylated (P) kinases tested using a multiplex immunoassay. Highlighted (yellow) P-proteins were upregulated, though not significantly, after a high dose of IL-6 treatment (16,000 pg/mL) compared to control-treated AECs. **(B)** IL-6 treatment (16,000 pg/mL) of AECs showed increasing trends of upstream kinases activation, JNK (2.1-fold) and p38MAPK (1.9-fold), which contributed to the downstream activation of STAT1 (1.5-fold), ATF2 (1.5-fold), and HSP27 (1.5-fold) compared to control samples. *N* = 5; mean ± SEM medium florescent intensity. **(C,D)** A pathologic dose of IL-6 did not increase the inflammatory profile of AECs, as seen by no changes in MMP9 activation or GM-CSF production when compared to control or term labor IL-6 treatment (3,300 pg/mL). *N* = 5; mean ± SEM.

A kinase immunoassay was performed to assess IL-6 concentrations within amniotic fluid from infectious pPROM samples (16,000 pg/mL) to determine if pro-labor pathways were activated (Figure 3). Pathologic and physiologic (3,300 pg/mL) IL-6 levels both showed increasing trends of upstream kinases JNK and p38MAPK activation, and the downstream kinase ATF2, compared to control AECs (Figures 3A,B). However, pathologic doses of IL-6 also showed increasing trends of downstream kinases STAT1 and HSP27 upregulation compared to control AECs (Figures 3A,B). Regardless of IL-6 treatment, AECs did not activate or increase expression of the pro-inflammatory mediators MMP9 and GM-CSF (Figures 3C,D).

To further confirm the contribution of the pPROM IL-6 level contributing to a labor phenotype, we tested the effect of IL-6 on AEC cell cycle, cell death, cell aging, and cellular transition.

### IL-6 Concentration Mimicking Infectious and Inflammatory pPROM Does Not Induce Changes in Cell Cycle, Cellular Aging, Cell Death, or Cellular Transition in AECs

Treatment with 16,000 pg/mL IL-6, a concentration often seen in infection and inflammation associated pPROM, was used to determine if such a high dose IL-6 could induce AEC cellular pathologies compared to the term labor concentration (3,300 pg/ml). Similar to our observations reported above, cell cycle analysis determined that pathologic doses of IL-6 did not induce any changes in G0, G1, S phase, or G2 compared to control cells (Supplementary Figure S2).

**FIGURE 4:**
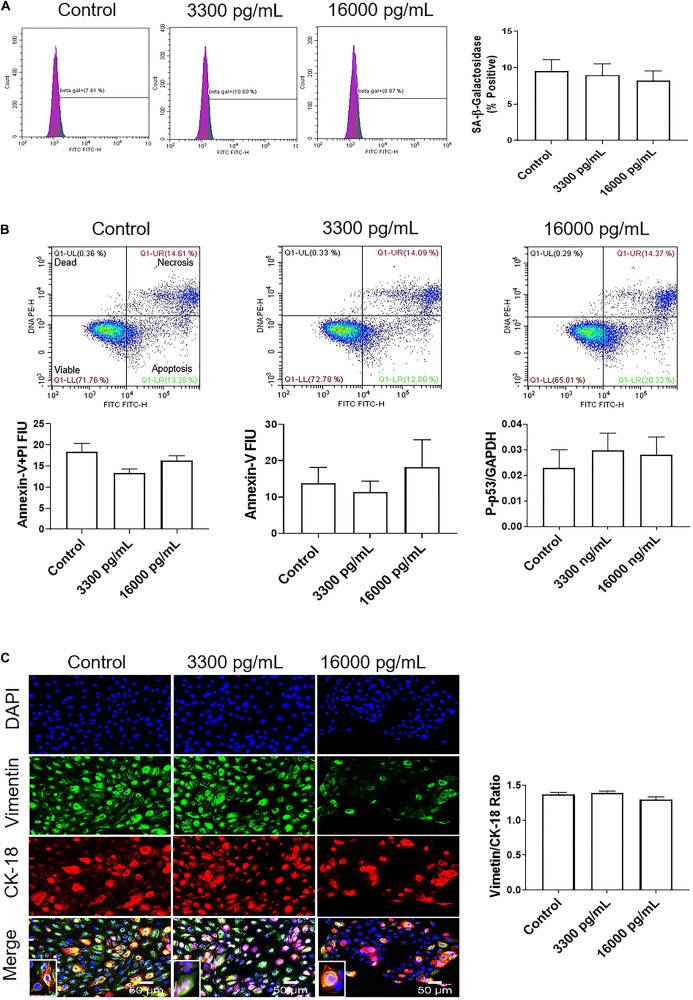
IL-6 concentration reported in the amniotic fluid of infection associated pPROM does not induce cellular aging, cell death, or cellular transitions in AECs. **(A)** Flow cytometry analysis of IL-6 induced senescence in AECs. Physiologic and pathologic concentrations of IL-6 did not increase the population of senescence-associated-β-galactosidase (SA-β-Gal) positive cells compared to controls. *N* = 5; mean ± SEM. **(B)** Flow cytometry analysis of necrosis and apoptosis of cells. Physiologic (3,300 pg/mL) and pathologic (16,000 pg/mL) concentrations of IL-6 did not induce necrosis, as indicated by a lack of changes in annexin-V + propidium iodide (PI) positive cells (top right box), nor apoptosis, as indicated by no changes in Annexin-V (bottom right box) expression between IL-6 treated and untreated cells. A protein kinase panel further confirmed a lack of apoptosis by showing that phospho-p53, a pro-apoptotic marker, did not change after treatment with IL-6. Fluorescence intensity units (FIU). *N* = 5; mean ± SEM. **(C)** Immunocytochemistry of intermediate filaments in AEC to assess cell transition. AECs were stained with both cytokeratin-18 (CK-18; epithelial marker) and vimentin (mesenchymal marker) to determine transition after physiologic and pathologic IL-6 treatment. Uniform intensity analysis was conducted on vimentin and CK-18 levels to derive the vimentin/CK-18 ratio. A high vimentin/CK-18 ratio indicates a mesenchymal phenotype, while a low ratio indicates an epithelial phenotype. IL-6 treatment did not change the vimentin/CK-18 ratio compared to control cells. Fluorescent images were captured at 20×. Red: cytokeratin-18 (CK-18), green: vimentin, blue: DAPI. *N* = 5; mean ± SEM. Scale bar 50 μm.

Additionally, flow cytometry indicated a lack of senescence (lack of change in SA-β-Gal activity), necrosis (lack of annexin-V + PI cells), or apoptosis (low annexin-V) (Figures 4A,B) compared to either 3,300 pg/ml or control. Lack of apoptosis was further validated by a protein kinase panel showing no change in phosphorylated-P53, a pro-apoptotic protein, regardless of the dose of treatment (Figure 4A). Given that our data show that IL-6 did not induce changes in the cell cycle, cellular aging, or cell death, we next determined if IL-6 induced cellular transitions (EMT or MET) in AEC.

Treatment with pathologic levels of IL-6 did not significantly change the vimentin/CK-18 ratio compared to controls and maintained the AEC metastate (Figure 4C). When compared to the term labor concentration, 16,000 pg/mL of IL-6 did not increase mesenchymal marker vimentin and maintained the cells in a cuboidal morphology (see inset), suggesting that IL-6 could play a role in AEC homeostasis (Figure 4C) rather than EMT induced cellular pathologies as we hypothesized.

To determine if IL-6 induced EMT at term or preterm *in vivo*, we conducted experiments using an IL-6 knockout (KO) mouse model.

### IL-6 KO in C57B/6 Mice Does Not Affect EMT at Term

**FIGURE 5:**
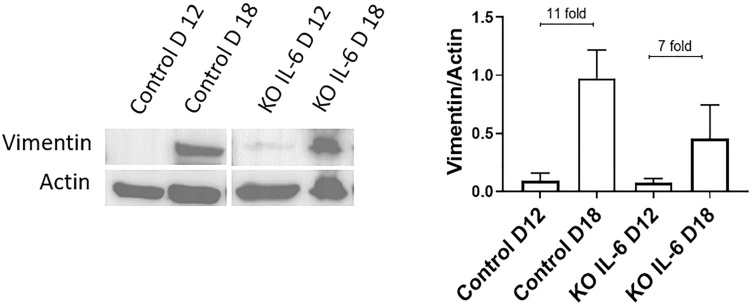
Normal and IL-6 KO mice undergo EMT associated changes at term. Western blot analysis and densitometry of mesenchymal marker vimentin in the amniotic sac of C57B/6 mice and KO IL-6 mice on day 12 (mid-gestation) and day 18 (term) of gestation. Amniotic sacs from control C57B/6 mice showed an increase in vimentin on day 18 compared to day 12 (11-fold), as previously document in CD1 models. The absence of IL-6 did not inhibit the increase in vimentin on day 18 compared to day 12 (7-fold). *N* = 3 for each category; mean ± SEM.

Recently, it was reported that human fetal membranes and mouse amniotic sacs both undergo senescence (Bonney et al., 2016; Polettini et al., 2018) as well as EMT at term labor (Richardson et al., 2020). Since the contribution of IL-6 is unclear for these two events, we utilized an established IL-6 knockout (KO) mouse (Gomez-Lopez et al., 2016) model to determine if IL-6 was needed to induce EMT at term in C57B/6 mice. Western blot analysis and densitometry of the mesenchymal marker vimentin were conducted on amniotic sacs of control C57B/6 mice and KO IL-6 mice on day 12 (D12) (mid-gestation) and day 18 (D18) (term) of gestation (Figure 5A). Amniotic sacs from control and IL-6 KO C57B/6 mice showed an 11- and 7-fold increase in vimentin on D18 compared to D12 (Figure 5A), respectively, as previously document in CD1 models (Richardson et al., 2020), suggesting the development of a terminal state of EMT as seen in human membranes at term labor. The absence of any significant changes in EMT marker expression in IL-6 KO mice suggests that IL-6 does not control the induction of EMT at term.

## Discussion

IL-6, often considered a pro-inflammatory cytokine, is one of the most studied cytokines in human parturition as well as in adverse pregnancy conditions (Menon et al., 2011). IL-6 shows constitutive expression in the uterine tissues during gestation and is elevated during parturition (Romero et al., 1990; Santhanam et al., 1991; Keelan et al., 1997; Lee et al., 2011; Devi et al., 2015); however, its exact function in different uterine compartments is still unclear. Irrespective of etiologies, IL-6 concentration is increased in various biological compartments in PTB and pPROM. IL-6 is also reported to increase prostaglandin productions from AECs and often referred in the literature as a “causal factor” for various adverse outcomes, specifically infection and inflammation associated PTB and pPROM (Romero et al., 1990; Santhanam et al., 1991). As our laboratory is interested in exploring the biology and function of fetal membrane cells as detailed in the introduction, we tested the fate of AECs, a key component of fetal membranes, in response to both physiologic and pathologic doses of IL-6 as a measure of its function. Using an *in vitro* AEC and *in vivo* mouse models, we report the following principal findings; (1) physiologic or pathologic concentrations of IL-6 increased cell signaling kinases (JNK, p38MAPK, ATF2, HSP27, c-JUN, MEK1, and STAT1) involved in various cellular functions, (2) activation of various signaling molecules by IL-6 did not result in the activation of inflammatory mediators, (3) IL-6 does not induce changes to the cell cycle, cellular aging (senescence), or cell death (necrosis and apoptosis), (4) IL-6 did not cause EMT in AECs and cells remained more epithelioid even at pathologic doses, suggesting that IL-6 is definitely not a pro-EMT cytokine in AECs, and (5) EMT was evident in the amniotic sac of mice on D18 compared to D12, confirming our prior reports (Richardson et al., 2020); however, in this study, IL-6 KO mice showed no difference in this pattern, suggesting that IL-6 does not impact the development of EMT at term.

Pregnancy and parturition involve a complex interplay between various endocrine, paracrine, and autocrine systems to maintain homeostasis. Disruptions to any of these processes will lead to adverse pregnancy outcomes such as PTB and pPROM, which are associated with ∼10.5% of all pregnancies around the globe (Blencowe et al., 2012, 2013; Lawn et al., 2013). Endocrine mediators such as progesterone are involved in pregnancy maintenance and parturition (Kota et al., 2013). Similarly, cytokines are a large class of secretory proteins implicated as endocrine, paracrine, and autocrine signaling mediators. Several cytokines have been functionally linked to various biological processes that maintain pregnancy and promote parturition. The most discussed cytokine is IL-6, as it is often reported as a biomarker and implicated in pathological pathways leading to adverse pregnancy outcomes (Romero et al., 1990; Santhanam et al., 1991; Chaemsaithong et al., 2016a). Lack of disruptive roles in cell cycle and cell death, it is likely that IL-6 is involved in pregnancy maintenance although the precise mechanistic functions, if any, are yet to be determined. Additionally, IL-6 could play a role with other cytokines, endocrine, paracrine, and autocrine signaling mediators to induce responses in AECs that were not evaluated in this study.

We were specifically interested in the role of IL-6 in cellular transitions, as this is one of the key mechanisms that recycles cells during gestation. Recent findings support the concept that AECs are constantly shed from the surface of the membrane and do not have a uniform surface (Richardson et al., 2017b, c). As the membranes grow, AECs undergo replicative senescence and become inflamed (Menon et al., 2013; Bredeson et al., 2014; Dixon et al., 2018; Hadley et al., 2018). These cells are often seen in the amniotic fluid and have been used in the past for cytogenetic analysis. Local inflammation in the membrane and increases in cytokines such as TGFβ (Richardson et al., 2018) can force them to transition into AMCs (Richardson et al., 2019, 2020). AMCs are highly mobile and find their way into the ECM through the recently reported microfractures in the membranes (Richardson et al., 2017c). As a part of the membrane remodeling process, AMCs are recycled and transitioned back to AECs (Richardson et al., 2019). This is mainly driven by progesterone through its membrane receptor 2 (PGRMC2) and mediated by c-MYC activation (Richardson et al., 2020). Cellular recycling, along with localized inflammation that rebuilds the ECM, helps to maintain membrane integrity. Reduced AMC recycling and their subsequent accumulation in the matrix occur at term prior to labor due to a “functional P4 withdrawal” (Richardson et al., 2020). This process is specific to the membranes and is due to PGRMC2 downregulation and not due to a change in phosphorylation status, as has been reported in the myometrium. OS at term and infection/inflammation markers can also force the downregulation of PGRMC2-mediated P4 withdrawal, creating a terminal state of EMT (Richardson et al., 2020). Since IL-6 is available at a baseline level in the amnion membranes under normal conditions (Keelan et al., 1997) and IL-6 increases following stimulation of any kind, we were specifically interested in its contribution to determining amnion membrane cell fate. Our data suggest that while IL-6 may still be a reliable marker for infection/inflammation in PTB and pPROM, it does not functionally contribute to the disruption of cellular homeostasis in the membranes.

Based on our data, both physiologic and pathologic doses of IL-6 showed a tendency to maintain the epithelial state, suggesting that endogenous IL-6 may play a role in maintaining the metastate of the cell rather than promoting any type of transition. We confirmed this hypothesis using an *in vivo* IL-6 KO mouse model. Analysis of amniotic sacs from IL-6 KO mice further confirmed our findings as the loss of IL-6 did not affect the expression of transition markers. These data suggest that, during a healthy pregnancy, baseline levels of IL-6 are an indicator or by-product of an ongoing inflammatory process; IL-6 may perhaps support pregnancy maintenance but not necessarily directly contribute to any of these functions. IL-6 activated various signaling molecules such as JNK, p38MAPK, MSK1, STAT1, HSP27, c-JUN, and ATF2, confirming its activity in AECs. Downstream targets of these signaling molecules include pro-inflammatory cytokines, prostaglandins, and matrix remodeling enzymes (Pirianov et al., 2015; Menon and Papaconstantinou, 2016; Cutler et al., 2017). In our study, activation of these signaling molecules by IL-6 did not result in a pro-inflammatory environment, suggesting that IL-6 alone, at doses that are often associated with normal and abnormal pregnancies, is not capable of inducing a pro-parturition status.

As mentioned above, IL-6 is a well-reported biomarker of inflammation and intraamniotic infection (Combs et al., 2015). Point of care (POC) tests using specific concentrations of IL-6 have been developed, and some are in clinical use (Chaemsaithong et al., 2015, 2016a,b; Kacerovsky et al., 2018). POC tests primarily use amniotic fluid to diagnose the status of MIAC and IAI and assist clinicians to provide better management in cases with preterm labor and or pPROM (Chaemsaithong et al., 2015, 2016a,b; Kacerovsky et al., 2018). These studies showed ethnic and other regional variations in IL-6 concentrations (Graham et al., 2018), and standard of care has been developed using population-specific values of IL-6 seen in amniotic fluids. Based on our data, the value of IL-6 as a biomarker does not change, as tissues respond to various stressors with substantial production of IL-6 indicative of an underlying pathology. However, this increase may not be indicative of any specific risk exposure or a pathobiological problem with either the mother or the fetus. Along with IL-6, additional biomarkers indicative of specific risks may be needed to provide risk-targeted intervention. In summary, IL-6 is not an indicator of changes related to any specific pathologic or physiologic function, but it is a good indicator of an overall disturbance during pregnancy that can alert clinicians to design management strategies. Sadowsky et al. (2006) has shown that an infusion of IL-6 into pregnant non-human primates does not cause preterm labor, whereas IL-1β and TNFα are better inducers of labor in this model.

We have earlier shown in AECs that IL-6 is functionally a very complex cytokine and performs its functions either through classical or *trans*-signaling mechanisms, which determine its biological activities (Noda-Nicolau et al., 2018). Classical signaling, through the membrane-bound IL-6 receptor (mIL-6R), produces an anti-inflammatory effect; conversely, *trans*-signaling through the soluble IL-6 receptor (sIL-6R) generates a pro-inflammatory effect (Noda-Nicolau et al., 2018). In both cases, the binding between IL-6 and mIL-6R or sIL-6R is followed by homodimerization of the β-subunit of glycoprotein 130 (gp130) (Lee et al., 2011; Rose-John, 2012), the signal transduction subunit. However, soluble gp130 (sgp130) functions as a natural antagonist of IL6/sIL6R *trans*-signaling, neutralizing IL-6 bioactivity by blocking IL-6/sIL-6R complex binding to gp130 and suppressing inflammation (Noda-Nicolau et al., 2018). We have tested the role of IL-6s in polymicrobial infection (genital mycoplasmas and *Gardnerella vaginalis*) of the fetal membranes (Noda-Nicolau et al., 2016). Our study reported that genital mycoplasmas alone, or in combination, inhibited IL-6 *trans*-signaling with increased sgp130 production. *G. vaginalis* activated the classical IL-6 signaling pathway (Noda-Nicolau et al., 2018). Polybacterial treatment resulted in a balanced response with neither pathway being favored. This study did not examine changes associated with IL-6 receptors that may play a role in mediating IL-6 functions.

We modeled our study using AECs, and the data and discussion are restricted to the function of IL-6 on these cells. Fetal membrane matrix also contains innate immune cells (∼2%) and our study did not test IL-6 and its receptors in these cells. AECs are constantly exposed to rapid changes in the neighboring environment during pregnancy as they are the innermost lining of the amniotic cavity and are constantly bathed in amniotic fluid (Richardson et al., 2017a; Menon et al., 2018). These changes in the environment include, but are not limited to, the redox state, endocrine factors, cytokines, chemokines and growth factors, feto-maternal immune cells, and extracellular vesicles carrying various cargoes that can also induce functional changes (Menon, 2019). Besides these biochemicals, mechanical stretching (due to growing fetal and amniotic fluid volume) and fetal movements can also impact amnion membrane cells (Joyce et al., 2016). Therefore, the impact on these cells provides valuable information on their behavior when in an environment with normal as well as high dose of IL-6. This is also a limitation of this study, as IL-6 may have other functional roles in other cell types in the uterine cavity, specifically in AMCs and the choriodecidua layer. AMCs residing in the stroma, are very reactive to pro-inflammatory stimulants; however, in the absence of rupture, any pro-inflammatory function of IL-6 maybe muted by AEC prior to that reaching AMC or other layers of the membranes. Additionally, IL-6 is hypothesized to play an important role during infection induce chorioamnionitis by activating immune cells within the choriodecidua layer. However, none of these are shown in any reliable and reproducible *in vitro* or animal models and the functional contributions of IL-6 in other reproductive tissues still remain as a hypothesis.

The above discussion is based on our data in AECs and not necessarily reflect its function in other cells of the uterine cavity. We conclude that the functional contributions of IL-6 are rather muted in AECs during reproduction. In association with other factors (e.g., infiltrating innate immune cells during labor), IL-6 may still exert some functions, but to have its own contributions IL-6 should align with its membrane and soluble receptors. This complexity likely minimizes the impact of this cytokine on AECs or it may be performing a very specific function that is not yet clear. An increase in IL-6 production by fetal membranes and other feto-maternal tissues in response to infection is likely a non-specific innate response and not an indication of a functional mediator of any labor-inducing pathways. Regardless of its function or lack thereof, IL-6 can still be a non-specific indicator and a good biomarker in various pregnancy-associated conditions.

## Data Availability Statement

All datasets generated for this study are included in the article/Supplementary Material.

## Ethics Statement

This study protocol was approved by the Institutional Review Board at The University of Texas Medical Branch (UTMB) at Galveston, TX, United States, as an exempt protocol to use discarded placenta after normal term cesarean deliveries (UTMB 11-251). No subject recruitment or consenting was done for this study and no identifiers were collected. The animal studies involving mice [Wild type (Jackson laboratories, Bar Harbor, ME, United States) and IL-6 deficient C57BL/6 mice] were reviewed and approved by the University of Vermont’s Institutional Animal Care and Use Committee [AAALAC-approved (IACUC #19-018)].

## Author Contributions

CO and LR conducted experiments, performed data analysis, and drafted the manuscript. TK helped run western blots. GS helped with placental specimens. EB contributed IL-6 KO mouse tissues. RM conceived the project, designed the experiments, provided funding and helped with data analysis and interpretation, and prepared the manuscript. All authors contributed to the article and approved the submitted version.

## Conflict of Interest

The authors declare that the research was conducted in the absence of any commercial or financial relationships that could be construed as a potential conflict of interest.

## Supplementary Material

FIGURE S1 IL-6 induced dose-dependent STAT3 activation in primary decidua cells validating its functionality. **(A)** Decidual cells were treated with IL-6, with doses as reported in the literature that can cause STAT3 activation, to validate the recombinant and commercially available IL-6 used for the treatments in our study. Western blot analysis showed that IL-6 treatment increased the activation of the downstream transcription factor STAT3 at pathologic levels compared to control (2.7-fold). *N* = 3; mean ± SEM.

FIGURE S2 IL-6 does not induce cell cycle changes in AECs. Cell cycle analysis was performed by measuring the DNA content and used to distinguish between different phases of the cell cycle after a physiologic or pathologic dose of IL-6. Fluorescence intensity that directly correlated with the amount of DNA contained in a cell was measured by flow cytometry. Concurrent parameter measurements made it possible to discriminate between S (red), G2 (blue), and mitotic cells (SubG0 in purple and G0 in pink). **(A)** Physiologic doses of IL-6 mimicking early gestation (330 pg/mL), mid-gestation (1,650 pg/mL), and term labor (3,300 pg/mL) do not affect normal cell cycle progression at sub G0, G0, S phase, or G2. Fluorescence intensity units (FIU). *N* = 3; mean ± SEM. **(B)** Pathologic doses of IL-6 seen in the amniotic fluid of infectious pPROM do not affect normal cell cycle progression at sub G0, G0, S phase, or G2 when compared to control or term labor treatments. Fluorescence intensity units (FIU). *N* = 3; mean ± SEM.
