# Supplementary figures and images for: Interleukin (IL)-6: A Friend or Foe of Pregnancy and Parturition? Evidence From Functional Studies in Fetal Membrane Cells
Source: Front Physiol. 2020 Jul 24;11:891. doi: 10.3389/fphys.2020.00891 (PMC7397758; doi:10.3389/fphys.2020.00891)

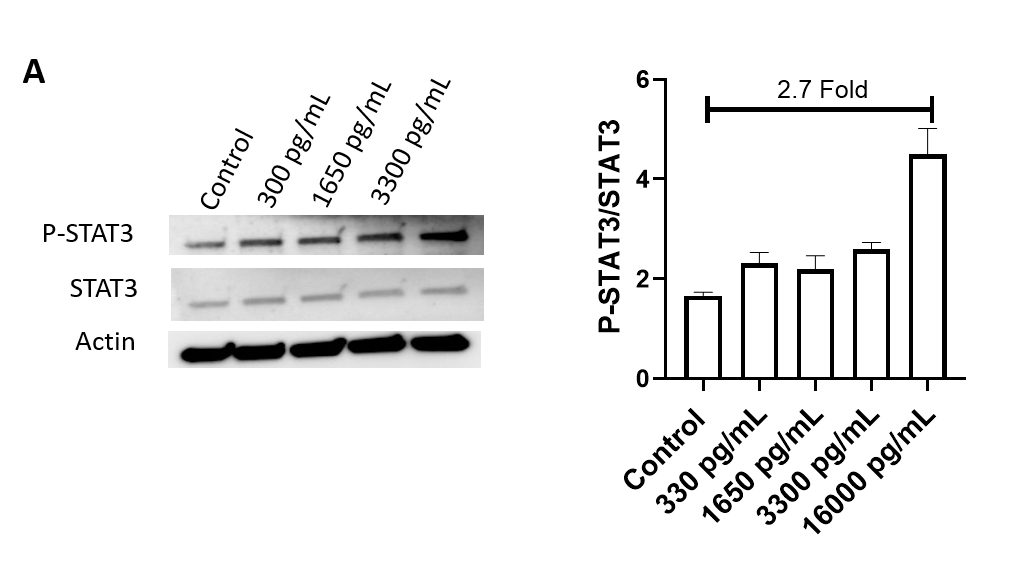

Supplement: FIGURE S1 — IL-6 induced dose-dependent STAT3 activation in primary decidua cells validating its functionality. (A) Decidual cells were treated with IL-6, with doses as reported in the literature that can cause STAT3 activation, to validate the recombinant and commercially available IL-6 used for the treatments in our study. Western blot analysis showed that IL-6 treatment increased the activation of the downstream transcription factor STAT3 at pathologic levels compared to control (2.7-fold). N = 3; mean ± SEM. [file Image_1.TIF]

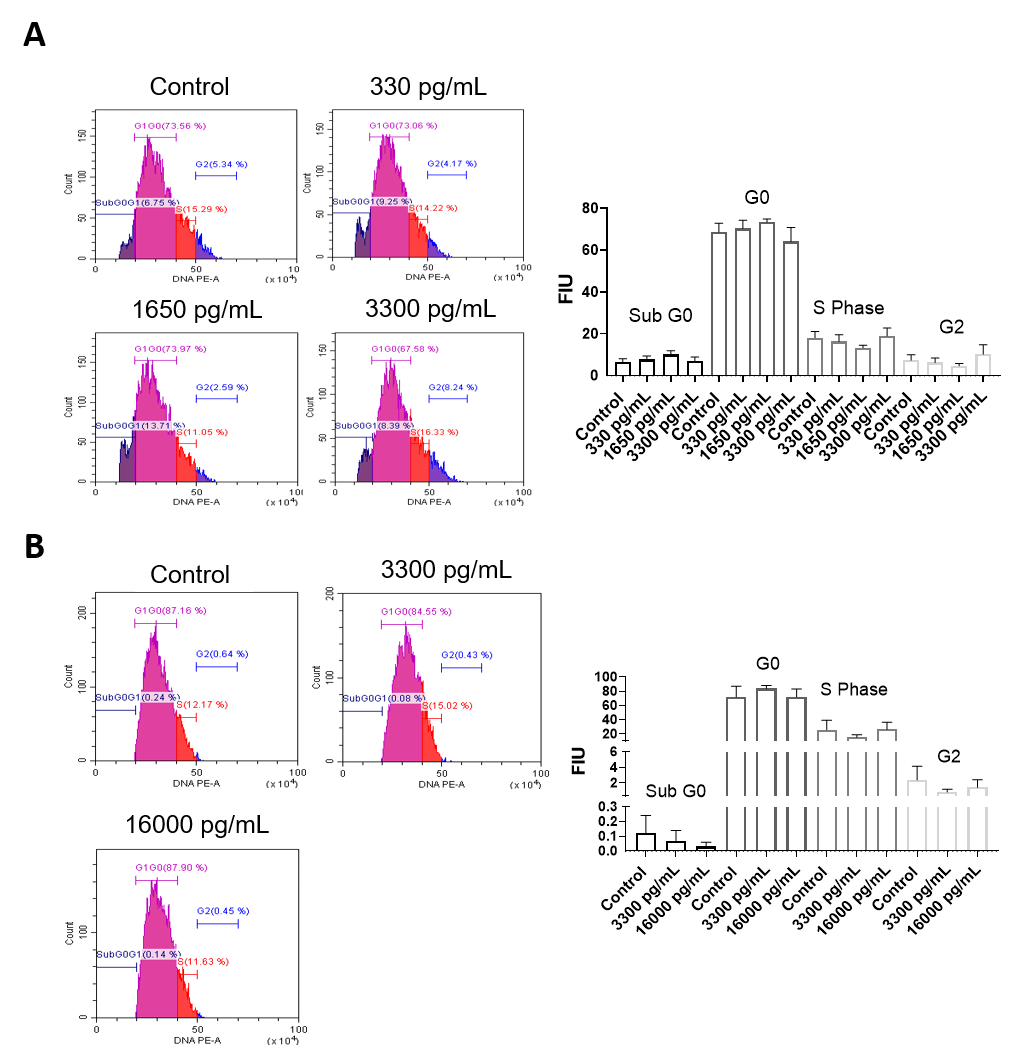

Supplement: FIGURE S2 — IL-6 does not induce cell cycle changes in AECs. Cell cycle analysis was performed by measuring the DNA content and used to distinguish between different phases of the cell cycle after a physiologic or pathologic dose of IL-6. Fluorescence intensity that directly correlated with the amount of DNA contained in a cell was measured by flow cytometry. Concurrent parameter measurements made it possible to discriminate between S (red), G2 (blue), and mitotic cells (SubG0 in purple and G0 in pink). (A) Physiologic doses of IL-6 mimicking early gestation (330 pg/mL), mid-gestation (1,650 pg/mL), and term labor (3,300 pg/mL) do not affect normal cell cycle progression at sub G0, G0, S phase, or G2. Fluorescence intensity units (FIU). N = 3; mean ± SEM. (B) Pathologic doses of IL-6 seen in the amniotic fluid of infectious pPROM do not affect normal cell cycle progression at sub G0, G0, S phase, or G2 when compared to control or term labor treatments. Fluorescence intensity units (FIU). N = 3; mean ± SEM. [file Image_2.TIF]
